# Genome Sequences of the Mycobacteriophages Awesomesauce and LastJedi

**DOI:** 10.1128/MRA.00707-20

**Published:** 2020-08-20

**Authors:** Madison E. Davis, Véronique A. Delesalle, Kayla D. Blankenship, Jeffrey T. Good, Clayr M. Kroenke, Gabrielle M. Kuba, Sean P. Sweeney, Christine A. Byrum

**Affiliations:** a Department of Biology, College of Charleston, Charleston, South Carolina, USA; b Department of Biology, Gettysburg College, Gettysburg, Pennsylvania, USA; Queens College

## Abstract

Bacteriophages Awesomesauce and LastJedi infect Mycobacterium smegmatis mc^2^155. While the Awesomesauce genome is 57,054 bp with 94 protein-coding genes, the LastJedi genome is 55,149 bp with 94 protein-coding genes. Nucleotide sequence comparison in Phamerator detected synteny between Awesomesauce gp49 to gp61 and singleton LilSpotty. Whole-genome BLASTn alignments revealed that LastJedi strongly resembles Clifton (99.41% identity).

## ANNOUNCEMENT

Bacteriophage Awesomesauce and LastJedi genomes were examined in collaboration with the Science Education Alliance-Phage Hunters Advancing Genomics and Evolutionary Science (SEA-PHAGES) program in efforts to characterize viral diversity. Both bacteriophages infect Mycobacterium smegmatis mc^2^155, a prominent acid-fast Gram-positive soil bacterium. Awesomesauce inhabited damp, shaded soil at Brown University (41.824593N, 71.40338W), while LastJedi was from mud near shrubs at the State University of New York College at Old Westbury (40.798447N, 73.57308W). Both were isolated (with enrichment at 23 to 24°C), purified, and amplified in M. smegmatis mc^2^155, as described in the SEA-PHAGES Discovery Guide (https://seaphagesphagediscoveryguide.helpdocsonline.com/home). The bacteriophages form clear plaques (∼1 mm), exhibit *Siphoviridae* morphology (images are available at https://phagesdb.org/phages/Awesomesauce and https://phagesdb.org/phages/LastJedi), and are F cluster/F1 subcluster members (1).

Following DNA extraction (Promega Wizard DNA clean-up system), sequencing libraries were produced (NEBNext Ultra II DNA library prep kit) and sequencing on an Illumina MiSeq system (MiSeq reagent kit v3) (2) was performed by the Pittsburgh Bacteriophage Institute, producing 1,068,099 and 636,029 single-end 150-bp reads for Awesomesauce and LastJedi, respectively. Reads were assembled into single contigs with Newbler v2.9 (3), and Consed v29 (4) was used to check accuracy and completeness and to identify the 3′ cohesive termini (2). Annotation was performed with the PECAAN workflow tool (5), and completed files were transferred to DNA Master v5.22.23 (https://phagesdb.org/DNAMaster). PECAAN resources applied to identify genes included Glimmer v3.0 (6), GeneMark v3.25 (7), Phamerator (8), Starterator v1.1 (https://seaphages.org/software), ARAGORN v1.2.38 (9), and tRNAscan-SE v3.0 (10). Functional assignments were made using BLASTp v2.9 (11), HHpred (12), TMHMM2 (http://www.cbs.dtu.dk/services/TMHMM), TOPCONS v2 (13), and the NCBI Conserved Domain Database (14). All programs utilized default settings. Table 1 summarizes additional findings.

**TABLE 1:** Characteristics of the Awesomesauce and LastJedi genomes

Phage name	GenBank accession no.	SRA accession no.	Genome size (bp)	GC content (%)	Coverage (×)	3′ extensions	No. of coding sequences	No. of tRNAs	Capsid size (nm)	Tail length (nm)
Awesomesauce	MT553347	SRX7690242	57,054	62.9	2,640	CCGAAGGCAT	94	1	89.35	249.0
LastJedi	MT553345	SRX7690245	55,149	61.8	1,652	CGGACGGCGC	94	0	84.3	277.0

In both genomes, key structural and assembly genes are on the left arm and nonstructural coding sequences are on the right arm. On the left arm, both genomes encode small and large terminase subunits, portal protein, capsid maturation protease, scaffolding protein, major capsid protein, head-to-tail adaptor, head-to-tail stopper, tail terminator, major tail protein, tail assembly chaperones, tape measure protein, minor tail proteins, lysin A/lysin B, and DNA polymerase III subunit. On the right arm, both code for genes regulating lysogeny (tyrosine integrase, immunity repressor, Cro, antirepressor, and excise protein), as well as WhiB family transcription factor, mobile element MPME1, and two glycosyltransferases.

Whole-genome BLASTn alignments (11) revealed that Awesomesauce is most similar to F1 mycobacteriophages TootsiePop (GenBank accession number MH020243) (99.34% identity, with 98% coverage), Misha28 (MH020242) (99.34% identity, with 98% coverage), and Piper2020 (MK494120) (97.76% identity, with 88% coverage). The Awesomesauce genome also includes Cas4-like exonuclease (gp66), two HNH endonucleases (gp1 and gp74), and two DNA methylases (gp73 and gp76). A rare synteny block (gp49 to gp61) that is highly conserved in 4 of the other 169 F1 mycobacteriophages (TootsiePop, Misha28, Piper2020, and Frankie [MG812488]) was identified. BLASTn comparison revealed remarkable colinearity and sequence similarity (98% identity) between the Awesomesauce synteny block and gp49 to gp61 in the singleton LilSpotty (MK977707), suggesting a common origin for the two. Excise (gp48) and MPME1 (gp62) border the synteny block and may facilitate its transposition. It was also noted that homologues to Awesomesauce gp82 (function unknown) occurred in 13 subcluster A1 bacteriophages but only 2 F1 mycobacteriophages (TootsiePop and Misha28) in addition to LilSpotty, suggesting shared origins for A1 and F1 bacteriophages.

LastJedi is highly homologous to F1 bacteriophages Clifton (GenBank accession number MH371115; 99.41% identity, with 95% coverage) and Brocalys (KU865302; 99.05% identity, with 95% coverage). Notable features include a toxin/antitoxin system (gp35 and gp36), two helix-turn-helix DNA binding domains (gp53 and gp54), Ku-like double-stranded DNA break-binding protein (gp57), AAA-ATPase (gp58), HNH endonuclease (gp59), DNA helicase (gp60), and two GIY-YIG endonucleases (gp65 and gp82).

### Data availability.

The Awesomesauce and LastJedi genomes are available in DDBJ/ENA/GenBank under accession numbers MT553347 and MT553345, respectively. Raw reads for Awesomesauce and LastJedi appear in the SRA under accession numbers SRX7690242 and SRX7690245, respectively. Note that in the Actinobacteriophage Database (https://phagesdb.org) (15), MargotMini (found by Jillian Nissen) is a duplicate of LastJedi.
